# Novel cardiac extracellular matrix biomarkers in STEMI: Associations with ischemic injury and long-term mortality

**DOI:** 10.1371/journal.pone.0302732

**Published:** 2024-05-13

**Authors:** Simon Andrup, Geir Ø. Andersen, Pavel Hoffmann, Jan Eritsland, Ingebjørg Seljeflot, Sigrun Halvorsen, Maria Vistnes

**Affiliations:** 1 Institute of Clinical Medicine, University of Oslo, Oslo, Norway; 2 Department of Cardiology, Oslo University Hospital Ullevål, Oslo, Norway; 3 Department of Cardiology, Section for Interventional Cardiology, Oslo University Hospital, Oslo, Norway; 4 Department of Cardiology, Center for Clinical Heart Research, Oslo University Hospital Ullevål, Oslo, Norway; 5 Institute for Experimental Medical Research, Oslo University Hospital and University of Oslo, Oslo, Norway; Gifu University School of Medicine Graduate School of Medicine: Gifu Daigaku Igakubu Daigakuin Igakukei Kenkyuka, JAPAN

## Abstract

**Background:**

We aimed to determine whether serum levels of proteins related to changes in cardiac extracellular matrix (ECM) were associated with ischemic injury assessed by cardiac magnetic resonance (CMR) and mortality in patients with ST-elevation myocardial infarction (STEMI).

**Methods:**

The concentrations of six ECM-related proteins (periostin, osteopontin, syndecan-1, syndecan-4, bone morphogenetic protein 7, and growth differentiation factor (GDF)-15) were measured in serum samples from patients on Day 1 and Month 4 after STEMI (n = 239). Ischemic injury was assessed by myocardial salvage index, microvascular obstruction, infarct size, and left ventricular function measured by CMR conducted during the initial admission (median 2 days after admission) and after 4 months. All-cause mortality was recorded after a median follow-up time of 70 months.

**Results:**

Levels of periostin increased from Day 1 to Month 4 after hospitalization, while the levels of GDF-15, osteopontin, syndecan-1, and syndecan-4 declined. At both time points, high levels of syndecan-1 were associated with microvascular obstruction, large infarct size, and reduced left ventricular ejection fraction, whereas high levels of syndecan-4 at Month 4 were associated with a higher myocardial salvage index and less dilatation of the left ventricle. Higher mortality rates were associated with periostin levels at both time points, low syndecan-4 levels at Month 4, or high GDF-15 levels at Month 4.

**Conclusions:**

In patients with STEMI, we found an association between serum levels of ECM biomarkers and ischemic injury and mortality. The results provide new insight into the role ECM components play in ischemic injury following STEMI and suggests a potential for these biomarkers in prognostication after STEMI.

## Introduction

In patients with ST elevation myocardial infarction (STEMI), the expansion of the infarct is a prognostic determinant for both mortality and heart failure [1]. The final size of the infarction is determined both by the ischemic area not salvaged by reperfusion and the injury caused to the reperfusion itself, i.e., the ischemia-reperfusion (IR) injury [2,3]. Since strategies to reduce ischemic injury might improve outcomes for STEMI patients, identification of prognostic biomarkers and enhanced understanding of the pathophysiological mechanisms involved in ischemic injury, is necessary to reveal novel management strategies in STEMI.

Detrimental changes in the cardiac extracellular matrix (ECM) seem to be involved in myocardial ischemic injury, which may contribute to the expansion of the infarcted area by inducing inflammation, creating microvascular dysfunction, and aggravating cardiac remodelling [4]. In the acute phase of myocardial infarction (MI), formation of a provisional ECM facilitates immune cell infiltration and activation of fibroblasts [5], while the ECM lining the vasculature is involved in coronary microvascular injury and obstruction [6]. At a later stage in the post-MI myocardium, accumulation of ECM not only replaces the necrotic cardiomyocytes in the infarcted area, but also creates fibrosis in the border zone and viable myocardium, resulting in a worsening of the cardiac function [7]. If the proteins involved in these ECM changes spill over to the circulation, they may serve as circulating markers of ischemic injury.

To identify biomarkers associated with ischemic injury, we quantified a panel of biomarkers related to ECM changes in serum samples from patients admitted to hospital with STEMI. We selected a panel of proteins known to be involved in inflammation, fibrosis, and ECM remodelling, related to the activity of transforming growth factor-β (TGF-β), and available in an appropriate assay. The selected markers were osteopontin [8], periostin [9], syndecan-1 [10], syndecan-4 [11], bone morphogenetic protein (BMP)-7 [12], and growth differentiation factor (GDF)-15 [13]. Since TGF-β is a critical regulator of post-infarction inflammation and fibrosis ECM remodelling [14,15], we hypothesized that these ECM-related proteins could be associated with the degree of ischemic injury and outcomes after MI. Indeed, detrimental clinical outcomes have been observed in patients with acute coronary syndromes and elevated circulating levels of GDF-15 [16], syndecan-1, periostin, and osteopontin [17–19], while increased levels of syndecan-4 have been observed in patients with MI [20]. However, there is limited knowledge about their association to myocardial ischemic injury. Ischemic injury was evaluated by cardiac magnetic resonance (CMR), including infarct size and left ventricular (LV) dimensions and function, as well as microvascular obstruction (MVO) and myocardial salvage index (MSI) as parameters for IR injury. Thus, the aim of the present study was to explore potential associations between the selected biomarkers measured in the acute and chronic phase after STEMI with 1) myocardial ischemic injury and cardiac function assessed by CMR imaging and 2) long-term mortality.

## Materials and methods

### Study population

The study population encompassed patients enrolled in the previously published “The Postconditioning in ST-Elevation Myocardial Infarction” (POSTEMI) trial [21]. Briefly, the study population consisted of 272 patients with a first time STEMI admitted to Oslo University Hospital Ullevål and treated by primary percutaneous coronary intervention (PCI) included between June 2009 and April 2012. Patients that were clinically unstable at admission, had a previous MI, or renal failure were excluded. The aim of the POSTEMI study was to investigate the effect of post-conditioning on IR injury, which proved to be neutral without significant effects on the primary endpoint of infarct size [21]. In this sub-study, we have used blood samples taken at Day 1 (median 14.6 (11.3–17.5) hours after PCI) and 4 months after PCI, in all patients that underwent CMR and had available samples on at least one of the two time-points. All-cause mortality was obtained from clinical records after a median time of 70 months follow-up [22]. The authors did not have access to information that could identify individual participants during or after data collection.

### Laboratory analyses

Serum samples obtained by centrifugation within 1 hour at room temperature at 2500 x *g* for 10 minutes and kept at -80°C, were used for quantification of osteopontin, periostin, syndecan-1, syndecan-4, GDF-15, and BMP-7 by the bead-based multiplex immunoassay Luminex (Magpix, BioRad, US). Serum samples were analysed in the spring of 2021, samples were thawed only once. The analyses were performed in accordance with the specifications of the manufacturer (R&D systems, Inc, US) and blinded for outcomes. Briefly, serum samples were diluted 1:2 and incubated with magnetic beads coated with capture antibodies for each biomarker, before adding detection antibodies and then streptavidin-phycoerythrin. The plate was analysed on a MAGPIX multiplex Reader (R&D systems, US). For values lower than the detection limit of the assay, a value corresponding to 50% of the 6th standard curve point was used (S1 Table). Intra-assay coefficients of variation where 10.9% for osteopontin, but between 1.5 and 3.5% for the other markers. Samples from the same individual were measured on the same plate.

### CMR protocol and image analysis

Patients were evaluated by CMR at a median of 2 days after admission for STEMI and then after 4 months, to determine the degree of ischemic and IR injury. The protocol has previously been described in detail [23]. Briefly, end-systolic and end-diastolic LV volumes and the corresponding left ventricular ejection fraction (LVEF) were obtained in short axis view. Late gadolinium enhancement was used to acquire final infarct size and MVO. Final infarct size was determined through manual tracing of the infarcted area’s contour across all short axis slices, whereas MVO was identified as the central dark region within the hyperintense infarcted area. T2-weighted images were employed to quantify the area at risk (AAR), defined as signal intensity exceeding 2 standard deviations from that of remote, non-ischemic myocardium, and delineated through manual tracing. MSI was calculated as [(area at risk—infarct size at 4 months)/area at risk] x 100, as previously described [23]. Adverse ischemic injury was defined as: Large infarctions (final infarct size in the ≥75^th^ percentile, as used previously[1] and corresponding to an infarct size >23% of LV mass in our material), reduced LVEF (<50% at Month 4), adverse LV remodelling (change in end-diastolic volume (EDV) ≥10% as suggested previously [24]), a low MSI (lowest third percentile based on histogram inspection), and a large AAR (>50% based on histogram inspection).

### Statistical analyses

Data are presented as number (%) for categorical data, while continuous data are presented as means ± standard deviation (SD) or median (interquartile range) depending on distribution. Test for normality was performed graphically with histograms and quantile-quantile-plots. Wilcoxon signed rank test was used to test for differences between biomarkers at Day 1 and Month 4, and Spearman’s test to examine the correlation between biomarkers and clinical variables. Survival data was analysed using the log rank test. Univariable and multivariable logistic regression analyses were performed with biomarkers and dichotomous outcomes for adverse ischemic injury. Covariates were selected based on clinical relevance and possible confounding effects for ischemic injury, and included age, time from symptom onset to balloon, post-conditioning status, and peak troponin T (model 1) and the addition of N-terminal pro-B-type natriuretic peptide (NT-proBNP) at Month 4 (model 2). For time-to-event statistics, Cox regression and Kaplan-Meier plots were computed. Biomarkers were further dichotomized into “high” and “low” levels using Liu’s method as described previously [25], where MVO was employed as the reference variable due to its central role in IR injury [6]. Predictability of biomarkers were assessed by receiver operating characteristics (ROC). Due to the explorative nature of the study, no formal test was used to correct for multiple comparisons. A two-tailed p-value of <0.05 was considered significant. All analyses were performed in STATA version 17.0 (College Station, StataCorp LLC, TX) to which the package cutpt [26] was installed for determining cut-points with Liu’s index. Graphs were produced using GraphPad Prism (version 9.3.1, GraphPad Software, CA).

## Results

### Baseline characteristics

The study population consisted of 239 STEMI patients with a mean age of 60 ± 11 years, and where 83% of the patients were male. Almost half of the patients were current smokers, while a minority had comorbidities like treated hypertension (27%) and treated diabetes mellitus (6%). The median peak troponin T value was 5848 ng/L. The POSTEMI trial excluded patients with renal failure defined as serum creatinine >200μmol/L, whereas 0.8% had a serum creatinine >150μmol/L (Table 1).

**Table 1:** Baseline characteristics of the included patients.

Characteristics	Total (n = 239)
Age (years)	60 ± 11
BMI (kg/m^2^)	26.6 (24.5, 29.1)
Male sex	199 (83)
Treated hypertension	65 (27)
Treated hypercholesterolemia	24 (10)
Diabetes mellitus	14 (6)
Current smoker	117 (49)
Time from symptom onset to balloon (minutes)	186 (125, 264)
Received post-conditioning	117 (49)
Left anterior descendent as culprit vessel	117 (49)
Circumflex branch as culprit vessel	27 (11)
Right coronary artery as culprit vessel	95 (40)
**Medications prior to infarction**	
Clopidogrel	0
Acetylsalicylic acid	11 (5)
Β-adrenoreceptor inhibitor	18 (8)
Angiotensin-converting enzyme or angiotensin II receptor inhibitor	42 (18)
Aldosterone antagonists	0
Diuretics	13 (5)
Warfarin	4 (2)
Amiodaron	0
Calcium-channel inhibitor	16 (7)
**Laboratory measurements**	
Peak troponin T (ng/L)	5848 (3300, 10473)
Peak C-reactive protein (mg/L)	20.3 (7.6, 46.3)
Serum creatinine at admission (μmol/L)	70 (62, 81)
NT-proBNP at admission (pg/ml)	76 (34, 186)

Data presented as mean ± SD, median (interquartile range), or numbers (%). BMI, body mass index; NT-proBNP, N-terminal pro-B-type natriuretic peptide.

### Temporal development of biomarkers levels

Levels of osteopontin, syndecan-1, syndecan-4, and GDF-15 declined from Day 1 to Month 4, with the most pronounced decline of ~30% for GDF-15 and osteopontin (Table 2). In contrast, levels of periostin increased by 34% from Day 1 to Month 4. For BMP-7, the majority of the samples were below the detection limit, and this biomarker was therefore excluded from further analyses. Only weak correlations (rho<0.4) between the measured biomarkers and clinical outcomes were detected (S1 Fig).

**Table 2:** Serum levels of biomarkers at Day 1 and Month 4.

Biomarker levels (ng/ml)	Day 1 (n = 228)	Month 4 (n = 231)	p-value
GDF-15	0.99 (0.72–1.34)	0.68 (0.53, 0.96)	**<0.0001**
Periostin	148 (105–206)	196 (144, 273)	**<0.0001**
Osteopontin	8.45 (4.77–14.85)	4.96 (2.36, 9.96)	**<0.0001**
Syndecan-1	2.74 (2.25–3.42)	2.60 (2.23, 3.06)	**0.0001**
Syndecan-4	2.47 (2.07–3.10)	2.25 (1.50, 2.84)	**<0.0001**

Data presented as median (interquartile range). p-values refer to comparisons of levels between Day 1 and Month 4 performed with Wilcoxon signed rank test. A p-value <0.05 was considered significant and marked in bold.

GDF, growth differentiation factor.

### Ischemic injury assessed by CMR

From Day 1 to Month 4, both mean LVEF and mean EDV increased by 6%, while the median infarct size at 4 months were 14% (IQR 8%– 23%) (Table 3). At least one adverse ischemic injury as defined by the dichotomized CMR parameters, i.e., presence of MVO, large infarct size, reduced LVEF, LV dilation, low MSI, or large AAR, occurred in 77% of the patients (n = 185) (Table 4).

**Table 3:** CMR parameters.

CMR parameters	Day 1	Month 4
LVEF (%)	50 ± 11	54 ± 12
EDV (ml)	170 ± 42	181 ± 54
ESV (ml)	86 ± 37	87 ± 48
Final infarct size (% of left ventricle)	–	14 (8, 23)
MVO (n)	116 (50)	–
Area at risk (% of left ventricle)	43 ±15	–
Myocardial salvage (%)	–	52 ± 22

Data presented as mean ± SD, median (interquartile range), or numbers (%). CMR, cardiac magnetic resonance; LVEF, left ventricular ejection fraction; EDV, end-diastolic volume; ESV, end-systolic volume; MVO, microvascular obstruction.

**Table 4:** Occurrence of adverse ischemic injury.

Adverse ischemic injury	n (%)
Presence of MVO	116 (50)
Large infarct size (infarct size ≥75^th^ percentile)	57 (25)
Reduction in systolic function (LVEF <50%)	64 (28)
Left ventricular dilation (increase in EDV >10%)	91 (40)
MSI in the lowest third percentile	67 (34)
Area at risk >50%	63 (31)

MVO, microvascular obstruction; LVEF, left ventricular ejection fraction; EDV, end-diastolic volume; MSI, myocardial salvage index.

### Associations between biomarker levels and ischemic injury assessed by CMR

**Fig 1:**
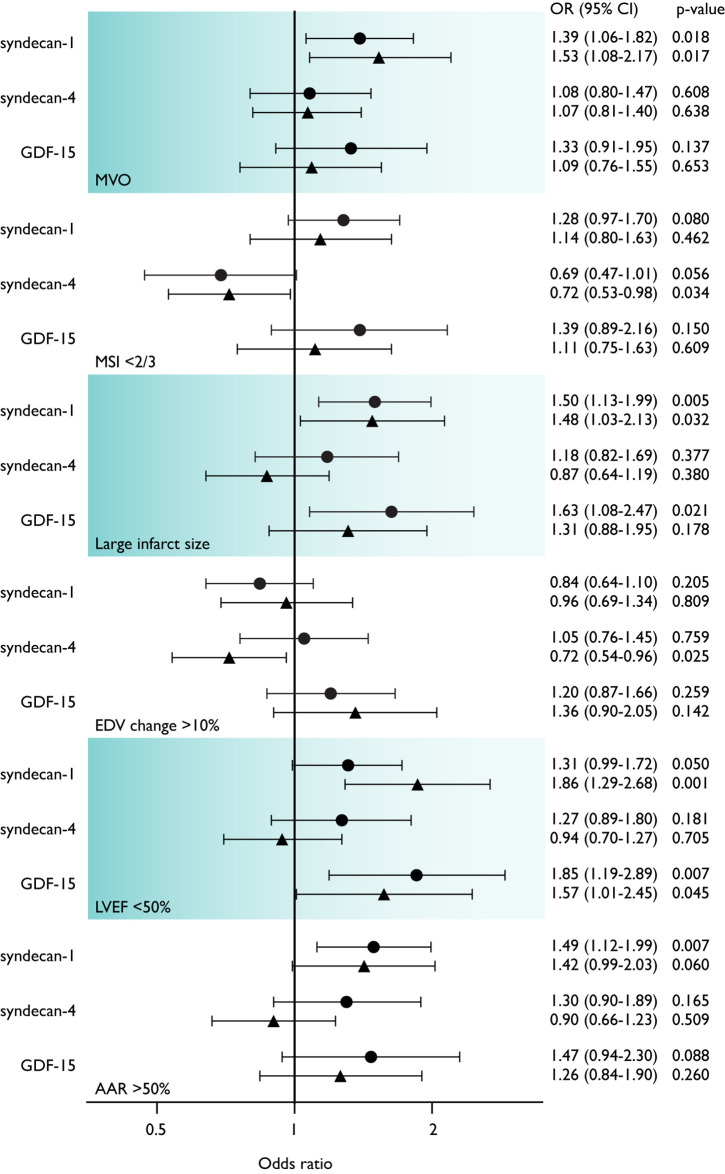
Logistic univariable regression analyses on dichotomized outcomes of ishemic injury and levels of biomarkers. Day 1 (circles) and Month 4 (triangles). Odds ratio (OR) per 1 ng/ml increase in concentration. MVO, microvascular obstruction; EDV, end-diastolic volume; LVEF, left ventricular ejection fraction; MSI, myocardial salvage index; AAR, area at risk.

Associations between biomarkers levels and adverse ischemic injury were determined by logistic regression. High levels of syndecan-1 at both time points were associated with the presence of MVO and large infarcts, while high syndecan-1 levels at Month 4 associated with reduced LVEF (Fig 1). For GDF-15, high levels at both time points were associated with reduced LVEF, while a high level at Day 1 correlated with a large infarct size (Fig 1). At Month 4, increased levels of syndecan-4 were associated with higher MSI and less LV dilation (Fig 1). The associations observed in univariable regression analyses (S3-S5 Tables in S1 File) did not remain significant after correcting for confounding factors in the predefined models.

### Associations between biomarker levels and mortality

We studied whether there was any association between biomarker levels and all-cause mortality (n = 20) by Cox proportional hazards models. In univariable analysis, higher levels of periostin on both time-points and GDF-15 at Month 4 were associated with an increased risk of all-cause mortality (Table 5), whereas a high level of syndecan-4 at Month 4 was associated with reduced risk of all-cause mortality (Table 5). These associations remained significant after adjusting for selected confounding factors (model 1). However, after including NT-proBNP levels (model 2), only the association between levels of GDF-15, periostin, and syndecan-4 at Month 4 and all-cause mortality remained significant (Table 5) (S6-S8 Tables in S1 File).

**Table 5:** Cox proportional hazards models for the association between biomarker levels and all-cause mortality.

	Univariable		Model 1		Model 2	
Biomarker	HR (95% CI)	p-value	HR (95% CI)	p-value	HR (95% CI)	p-value
GDF-15, Day 1	1.23 (0.93–1.64)	0.149	1.08 (0–77–1.52)	0.644	0.66 (0.42–1.05)	0.079
GDF-15, Month 4	1.59 (1.28–1.97)	**<0.0001**	1.82 (1.36–2.43)	**<0.0001**	1.45 (1.000–2.11)	**0.050**
Periostin, Day 1	1.006 (1.002–1.01)	**0.009**	1.005 (1.0004–1.01)	**0.034**	1.003 (0.998–1.008)	0.276
Periostin, Month 4	1.007 (1.003–1.01)	**0.001**	1.006 (1.002–1.01)	**0.003**	1.005 (1.0007–1.01)	**0.024**
Syndecan-4, Day 1	0.62 (0.36–1.07)	0.087	0.74 (0.42–1.29)	0.286	0.50 (0.23–1.08)	0.079
Syndecan-4, Month 4	0.48 (0.28–0.84)	**0.010**	0.48 (0.26–0.88)	**0.017**	0.44 (0.22–0.84)	**0.014**

All concentrations in ng/ml. Hazard ratios are calculated for per 1 unit increase in concentration. Model 1: Age, symptom-to-balloon time, post-conditioning status, peak troponin T. Model 2: model 1 + NT-proBNP at Month 4. P<0.05 were considered significant and marked in bold.

GDF, growth differentiation factor; NT-proBNP, NT-proBNP, N-terminal pro-B-type natriuretic peptide;HR, hazard ratio; CI, confidence interval.

### Prediction of all-cause mortality using biomarker cut-off levels

**Fig 2:**
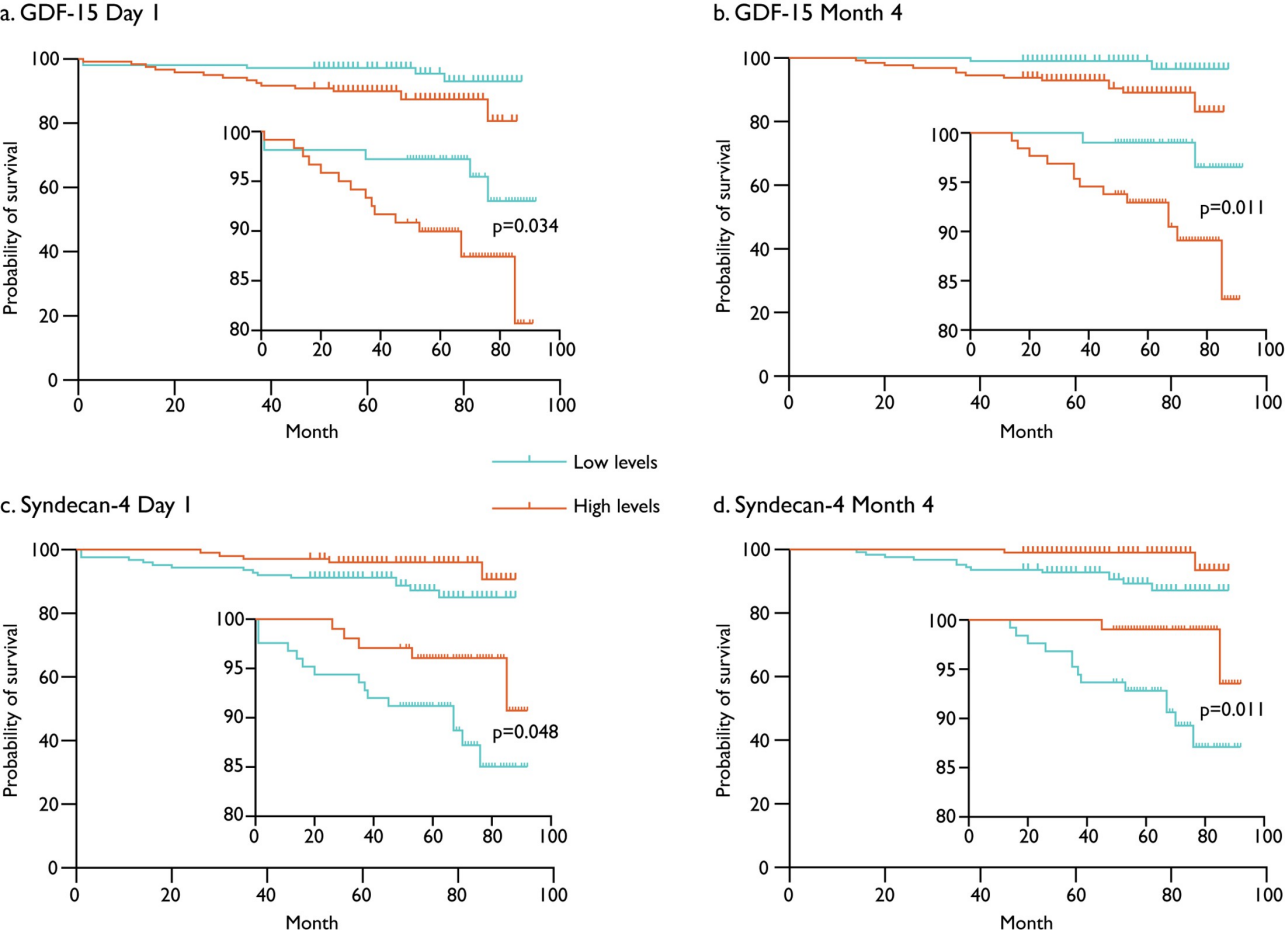
Kaplan-Meier plots on all-cause mortality and the dichotomized biomarkers GDF-15 and syndecan-4. Serum levels of GDF-15 at Day 1 (a), Month 4 (b), and syndecan-4 levels at Day 1 (c) and Month 4 (d) High (turquoise) and low (orange) serum levels determined with cut-off levels estimated by Liu’s method, while log-rank test was employed to compare the probability of mortality in high versus low serum levels. A p-value <0.05 was considered significant. GDF, growth differentiation factor.

To determine the associations between the outcomes in patients with high levels of the selected biomarkers, biomarker levels were dichotomized into high and low levels based on cut-off points for optimal detection of the presence of MVO (Table 6). We observed that the risk for all-cause mortality was increased in patients with high levels of GDF-15 (Day 1: p = 0.034; Month 4: p = 0.011) and decreased in patients with high levels of syndecan-4 (Day 1: p = 0.048; Month 4: p = 0.011) (Fig 2).

**Table 6:** Cut-off levels of biomarkers.

Biomarker	Cut-off level Day 1	Low (n (%))	High (n (%))	Cut-off level Month 4	Low (n (%))	High (n (%))
GDF-15	0.97	108 (47)	120 (53)	0.63	102 (44)	129 (56)
Periostin	160	129 (57)	99 (43)	167	87 (38)	144 (62)
Osteopontin	8.9	122 (54)	106 (46)	4.89	115 (50)	116 (50)
Syndecan-1	2.96	137 (60)	91 (40)	2.49	102 (44)	129 (56)
Syndecan-4	2.62	125 (55)	103 (45)	2.39	126 (55)	105 (45)

All concentrations in ng/ml. Cut-off levels calculated with Liu’s method.

### The performance of syndecan-4 and GDF-15 levels in prediction of all-cause mortality

**Fig 3:**
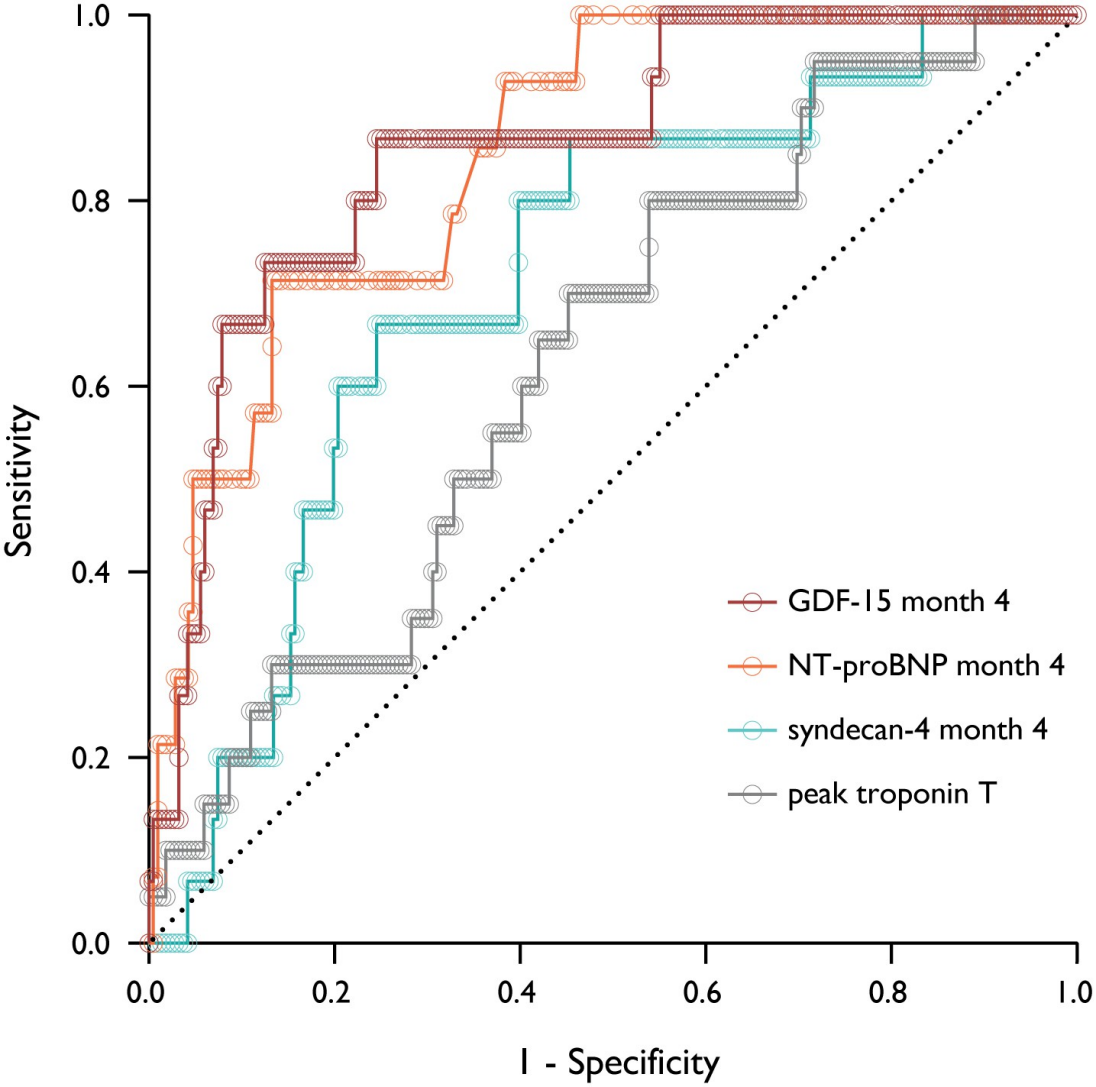
ROC curves for the prediction of all-cause mortality. Prediction based on the levels of GDF-15 at Month 4, syndecan-4 at Month 4, NT-proBNP at Month 4 and peak troponin T measured during the initial admission. ROC, receiver operating characteristics; AUC, area under the curve; GDF, growth differentiation factor; NT-proBNP, N-terminal pro-B-type natriuretic peptide.

We examined the performance of the biomarker levels to predict all-cause mortality based on ROC curves, where area under the curve (AUC) was calculated for biomarkers that were significant in the regression analyses. An ability to predict total mortality was demonstrated for levels of GDF-15 (AUC 0.86 (95% CI 0.76–0.95)) and syndecan-4 (AUC 0.72 (95% CI 0.59–0.84)) at Month 4, where the best prediction was observed for GDF-15 (p<0.0001 for difference against syndecan-4). Based on the AUCs, peak troponin T levels (AUC 0.63 (95% CI 0.51–0.75)) were inferior to GDF-15 (p = 0.006) and syndecan-4 (p = 0.003), while NT-proBNP levels at Month 4 (AUC 0.85 (95% CI 0.76–0.94)) were similar to GDF-15 (p>0.05) and superior to syndecan-4 (p<0.0001) (Fig 3, S2 Table in S1 File). Using combinations of the measured biomarkers did not improve the prediction of total mortality compared to the individual AUCs.

## Discussion

In this study, we found that circulating ECM biomarkers were associated with ischemic injury and long-term mortality in patients with STEMI. The major findings were that 1) high levels of syndecan-1 in the acute phase were associated with IR injury, 2) high levels of syndecan-4 at Month 4 after STEMI were associated with improved myocardial salvage, less LV dilation, and lower risk of death, 3) high periostin levels at both time points were associated with a higher risk of mortality, and 4) high levels of GDF-15, in particular at Month 4, were associated with both ischemic injury and mortality. To our knowledge, we are the first to demonstrate a temporal profile of several of these biomarkers after STEMI and to show an association to multiple parameters of ischemic injury assessed by CMR.

Wound healing and scar formation post-MI can be divided into phases of inflammation, proliferation, and maturation [7], where our samples taken at Day 1 and Month 4 correspond to the transition to the proliferative and maturation phase, respectively. In general, the observed reduction in biomarker levels from Day 1 to Month 4 may be explained by an initial increase of circulating concentrations due to tissue damage and subsequent acute inflammatory and fibrotic activity. Indeed, concentrations of syndecan-4 [27] and osteopontin [28] are reported to peak between Day 1 and 3 after MI, although later peaks are also reported for syndecan-4 [20]. Contrary to the other biomarkers, periostin levels increased during the initial four months, which can be explained by an early suppression post-MI as observed in other studies [29,30], with a later increase related to tissue remodelling. The time-dependent variation in the biomarker concentrations stress the need for standardized sampling time points and multiple measurements.

Our observations of an association between circulating ECM related biomarkers and adverse outcomes after STEMI adds new aspects to a growing body of evidence of their biomarker potential. Although associations between syndecan-1 and MVO [31] and mortality [17], and between periostin levels and major adverse cardiovascular events [18] have previously been reported in STEMI patients, we are the first to demonstrate that these associations are present beyond the acute phase. To the best of our knowledge, the association between syndecan-4 levels and outcomes after STEMI has not previously been studied. The favourable outcomes associated with high syndecan-4 levels in our study contrasts with other studies showing more incident MI [32], and poorer outcomes in high-risk individuals and other cardiovascular diseases [33–35] in patients with high serum levels. The divergent findings could stem from the varying roles of syndecan-4 in fibrosis, depending on the phase of disease progression and shedding [11]. Moreover, elevated levels may indicate patients with more advanced disease states, as evidenced in studies of heart failure patients where syndecan-4 levels negatively correlate with LVEF [36] However, when measuring syndecan-4 at specific time points following infarction as in our study, variations may mirror individual differences in syndecan-4 levels, potentially conferring protective effects. The prognostic value of GDF-15 in coronary disease, on the other hand, has been reported in numerous studies [37,38], where high levels consistently predict mortality from 30 days [39] up to 10 years [40] after MI, and correlates to ischemic injury [41]. However, fewer studies have examined GDF-15 levels at multiple time points. Of note, our finding that outcomes are more strongly associated with GDF-15 at Month 4, corresponds to a recent meta-analysis demonstrating a better prognostic ability when detected after the initial stabilization [16]. These observations indicate that GDF-15 is a better prognosticator when measured in the chronic phase, which provides useful information regarding a potential application of the biomarker. However, the lack of significance between biomarkers and ischemic injury after correction for confounding factors indicate that the incremental value of the biomarker levels may be limited for this potential clinical use.

Alterations in circulating levels of ECM biomarkers in patients with STEMI can reflect spill-over of accumulated ECM components, or actively released components. Previous studies showing upregulation of periostin in cardiac fibroblasts [14] and of osteopontin in cardiac macrophages [28], during MI indicate a cardiac origin for their elevated circulating levels. For GDF-15, on the other hand, its absence in fibrotic myocardium suggests other sources of circulating GDF-15 in heart disease [42]. Released peptides can also result from shedding, which denotes the protease-mediated release of the extracellular domain of syndecans. Interestingly, shedding of syndecan-4 may itself modulate ischemic injury, since shedding can result in liberation of cytokines and induction of fibrosis [11]. While the myocardium is a source for inflammation-induced shedding of syndecan-4 following MI [27], syndecan-1 is often shed from the vascular glycocalyx [17,43]. In summary, based on the known properties of these proteins, the circulating levels of the biomarkers probably depend on their tissue distribution, cellular sources, and mechanisms for release into the circulation.

The observed alterations in circulating levels support a pathogenic role of these ECM-related markers in post-MI remodelling and ischemic injury. Circulating syndecan-1 is suggested to reflect endothelial damage [17,43], where damaged endothelial glycocalyx may contribute to the vascular plugging that results in MVO [6]. Indeed, our observation of an association between serum syndecan-1 and larger infarcts supports a more aggravated IR injury in patients with higher syndecan-1 levels. Osteopontin and periostin, on the other hand, are involved fibrosis and the activation of fibroblasts [44]. In experimental studies, periostin contributes to detrimental fibrotic responses, as overexpression aggravates IR injury [45], and depletion reduces infarct size [46]. As such, the higher rate of adverse clinical outcomes in patients with high periostin levels, could be related to more advanced fibrosis in these cases. Syndecan-4 is oppositely linked to cardiac fibrosis in experimental studies with a transient myocardial upregulation protecting against fibrotic mechanisms following pressure overload [11], and where TGF-β activity is lowered by syndecan-4 overexpression [47] and aggravated by syndecan-4 knockout [48]. A protective effect on TGF-β-induced fibrosis could explain our observations of beneficial outcomes in patients with high syndecan-4 levels. Taken together, the circulating levels of periostin, syndecan-1, and syndecan-4 can be explained by known pathological roles in IR injury and cardiac fibrosis. GDF-15, on the other hand, possess no known functions in the cardiovascular system [49], although a higher circulating levels might reflect a larger degree of cellular senescence [50].

### Limitations

Although we have measured serum levels of the biomarkers at two time points, determination of a full temporal profile with peak values necessitates more frequent sampling. Only a few deaths occurred during the follow-up period, which limited the number of covariates used in regression analyses. Thus, associations between biomarkers, and outcomes can be affected by other confounding factors, such as sex [32,37]. Furthermore, we have refrained from measuring TGF-β directly, because circulating levels are less likely to reflect myocardial activity, due to its local activation in the tissue and high abundancy in platelets [51]. Non-cardiac sources may also contribute to the levels of the selected biomarkers in the circulation. Additionally, since our study utilized samples stored for up to 12 years, there is a possibility of alterations of protein concentrations over time. Last, as shed biomarkers may be proteolytically cleaved and therefore not contain the whole protein, as for syndecan-1 [17], syndecan-4 [27], and osteopontin [11], the assays to detect these biomarkers should ideally be characterized to determine which part of the protein that is detected.

### Future perspectives

To identify patients that may benefit from novel treatment strategies for cardioprotection, non-invasive assessment of ischemic injury and prognosis after STEMI is useful. The biomarkers examined in this study hold promise in uncovering additional disease processes beyond the currently utilized biomarkers of troponins and natriuretic peptides, which signifies cardiomyocyte necrosis and mechanical stretch, respectively [52]. Therefore, integrating biomarkers associated with ECM changes could allow a more comprehensive risk assessment for patients following MI. Identifying individuals at risk through such biomarker measurements could enable more precise preventive strategies, such as more intense treatment and follow-up. Thus, our results warrant further research on the use of GDF-15, periostin, syndecan-1, and syndecan-4 as biomarkers for ischemic injury and poor outcomes in patients with MI.

## Conclusion

In this study, we found an association between levels of ECM biomarkers and myocardial ischemic injury and mortality in STEMI patients, suggesting a role of ECM components in ischemic injury and in prognostication after STEMI.

## Supporting information

S1 Graphical abstract (TIF)

S1 Fig Spearman correlation heatmap. (TIFF)

S1 File Supplementary material. (DOCX)
