# Supplementary figures and images for: Novel cardiac extracellular matrix biomarkers in STEMI: Associations with ischemic injury and long-term mortality
Source: PLoS One. 2024 May 13;19(5):e0302732. doi: 10.1371/journal.pone.0302732 (PMC11090350; doi:10.1371/journal.pone.0302732)

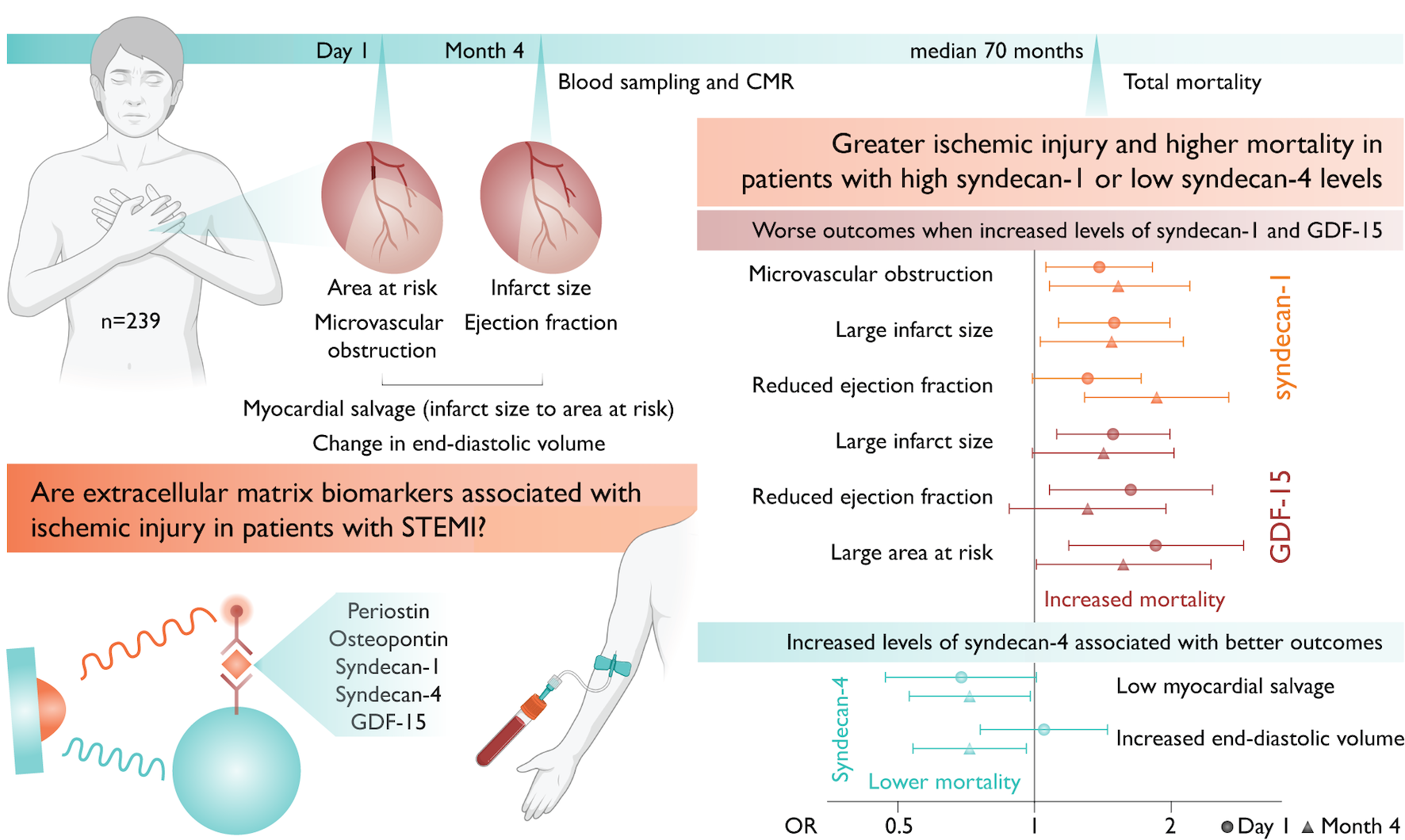

Supplement: S1 Graphical abstract — (TIF) [file pone.0302732.s001.tif]

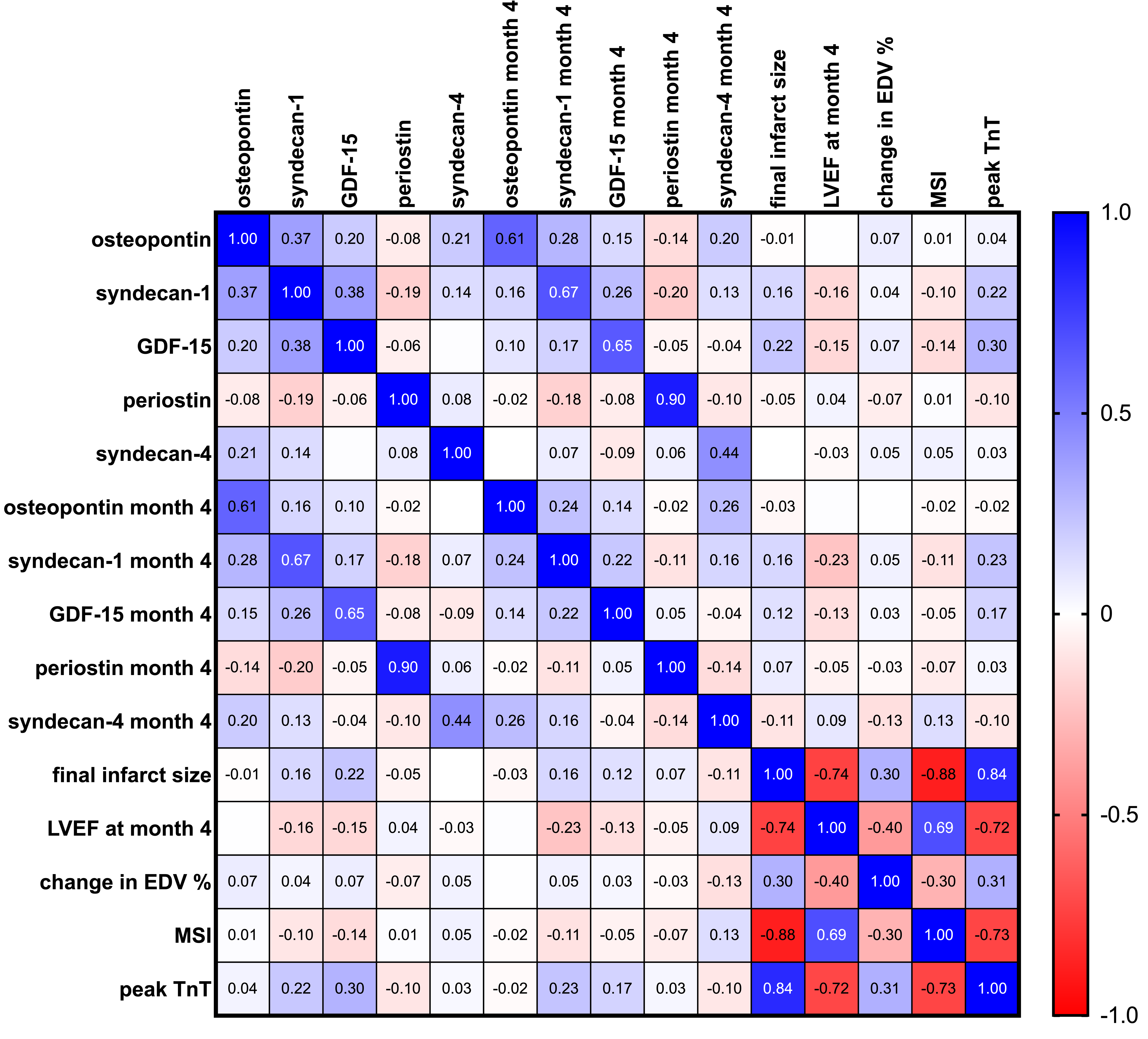

Supplement: S1 Fig — (TIFF) [file pone.0302732.s002.tiff]
