# Supplementary material for: Novel cardiac extracellular matrix biomarkers in STEMI: Associations with ischemic injury and long-term mortality
Source: PLoS One. 2024 May 13;19(5):e0302732. doi: 10.1371/journal.pone.0302732 (PMC11090350; doi:10.1371/journal.pone.0302732)
Supplement: S1 File — (DOCX) [file pone.0302732.s003.docx]

**Supplementary Material**

**Supplementary Results**

*Removal of outliers and limits of quantification*

One outlier for myocardial salvage index was removed before statistical analysis, due to a larger infarct size than AAR. For biomarker levels, limits of quantification in the Luminex assay are reported in Table S1.

Table S1. Limit of quantification of biomarker levels

| Biomarker | Lower limit of quantification | Upper limit of quantification | Out of range, n (%) |
| --- | --- | --- | --- |
| GDF-15 | 0.0057 | 4.3 | 0 (0) |
| Periostin | 1.2 | 406 | 0 (0) |
| Osteopontin | 1.1 | 381 | 9 (3) |
| Syndecan-1 | 0.073 | 28.3 | 0 (0) |
| Syndecan-4 | 0.013 | 10.6 | 0 (0) |

*All concentrations in ng/ml. Limits of quantification defined as averages of highest and lowest measured standard concentration. GDF, growth differentiation factor.*

Table S2. Univariable logistic regression analyses.

| **Biomarker** | **OR (95% CI)** | **p-value** | **Standard error** | **r^2^** |
| --- | --- | --- | --- | --- |
| **MVO** | | | | |
| Syndecan-1, Day 1 (ng/ml) | 1.39 (1.06–1.82) | **0.018** | 0.19 | 0.02 |
| Syndecan-1, Month 4 (ng/ml) | 1.53 (1.08–2.17) | **0.017** | 0.27 | 0.02 |
| Syndecan-4, Day 1 (ng/ml) | 1.08 (0.8­–1.47) | 0.608 | 0.17 | <0.01 |
| Syndecan-4, Month 4 (ng/ml) | 1.07 (0.81–1.4) | 0.638 | 0.15 | <0.01 |
| GDF-15, Day 1 (ng/ml) | 1.33 (0.91–1.95) | 0.137 | 0.26 | <0.01 |
| GDF-15, Month 4 (ng/ml) | 1.09 (0.76–1.55) | 0.653 | 0.2 | <0.01 |
| Periostin, Day 1 (ng/ml) | 1 (0.996–1.004) | 0.994 | <0.01 | <0.01 |
| Periostin, Month 4 (ng/ml) | 1 (0.998–1.003) | 0.605 | <0.01 | <0.01 |
| Osteopontin, Day 1 (ng/ml) | 1 (0.97–1.02) | 0.735 | 0.01 | <0.01 |
| Osteopontin, Month 4 (ng/ml) | 1 (0.97–1.03) | 0.948 | 0.01 | <0.01 |
| **Change in EDV >10%** | | | | |
| Syndecan-1, Day 1 (ng/ml) | 0.84 (0.64–1.1) | 0.205 | 0.12 | <0.01 |
| Syndecan-1, Month 4 (ng/ml) | 0.96 (0.69–1.34) | 0.809 | 0.16 | <0.01 |
| Syndecan-4, Day 1 (ng/ml) | 1.05 (­0.76–1.45) | 0.759 | 0.17 | <0.01 |
| Syndecan-4, Month 4 (ng/ml) | 0.72 (0.54–0.96) | **0.025** | 0.1 | 0.02 |
| GDF-15, Day 1 (ng/ml) | 1.2 (0.87–1.66) | 0.259 | 0.2 | <0.01 |
| GDF-15, Month 4 (ng/ml) | 1.36 (0.9–2.05) | 0.142 | 0.28 | <0.01 |
| Periostin, Day 1 (ng/ml) | 1 (0.997–1.004) | 0.664 | <0.01 | <0.01 |
| Periostin, Month 4 (ng/ml) | 1 (0.998–1.003) | 0.572 | <0.01 | <0.01 |
| Osteopontin, Day 1 (ng/ml) | 0.99 (0.97–1.02) | 0.674 | 0.01 | <0.01 |
| Osteopontin, Month 4 (ng/ml) | 1.02 (0.99–1.05) | 0.305 | 0.02 | <0.01 |
| **LVEF<50% at Month 4** | | | | |
| Syndecan-1, Day 1 (ng/ml) | 1.31 (0.99–1.72) | **0.050** | 0.18 | 0.02 |
| Syndecan-1, Month 4 (ng/ml) | 1.86 (1.29–2.68) | **0.001** | 0.35 | 0.04 |
| Syndecan-4, Day 1 (ng/ml) | 1.27 (0.89–1.8) | 0.181 | 0.23 | <0.01 |
| Syndecan-4, Month 4 (ng/ml) | 0.94 (0.7–1.27) | 0.705 | 0.14 | <0.01 |
| GDF-15, Day 1 (ng/ml) | 1.85 (1.19–2.89) | **0.007** | 0.42 | 0.04 |
| GDF-15, Month 4 (ng/ml) | 1.57 (1.01–2.45) | **0.045** | 0.36 | 0.02 |
| Periostin, Day 1 (ng/ml) | 1 (0.996–1.005) | 0.713 | <0.01 | <0.01 |
| Periostin, Month 4 (ng/ml) | 1 (0.999–1.005) | 0.169 | <0.01 | <0.01 |
| Osteopontin, Day 1 (ng/ml) | 0.999 (0.97–1.03) | 0.95 | 0.02 | <0.01 |
| Osteopontin, Month 4 (ng/ml) | 0.97 (0.93–1.01) | 0.13 | 0.02 | 0.01 |
| **Final infarct size >75th percentile** | | | | |
| Syndecan-1, Day 1 (ng/ml) | 1.5 (1.13–1.99) | **0.005** | 0.22 | 0.03 |
| Syndecan-1, Month 4 (ng/ml) | 1.48 (1.03–2.13) | **0.032** | 0.27 | 0.02 |
| Syndecan-4, Day 1 (ng/ml) | 1.18 (0.82–1.69) | 0.377 | 0.22 | <0.01 |
| Syndecan-4, Month 4 (ng/ml) | 0.87 (0.64–1.19) | 0.380 | 0.14 | <0.01 |
| GDF-15, Day 1 (ng/ml) | 1.63 (1.08–2.47) | **0.021** | 0.35 | 0.03 |
| GDF-15, Month 4 (ng/ml) | 1.31 (0.88–1.95) | 0.178 | 0.27 | <0.01 |
| Periostin, Day 1 (ng/ml) | 0.996 (0.992–1.001) | 0.133 | <0.01 | 0.01 |
| Periostin, Month 4 (ng/ml) | 1 (0.996–1.002) | 0.918 | <0.01 | <0.01 |
| Osteopontin, Day 1 (ng/ml) | 1 (0.97–1.03) | 0.91 | 0.02 | <0.01 |
| Osteopontin, Month 4 (ng/ml) | 0.98 (0.94–1.02) | 0.264 | 0.02 | <0.01 |
| **MSI<2/3** | | | | |
| Syndecan-1, Day 1 (ng/ml) | 1.28 (0.97–1.7) | 0.080 | 0.18 | 0.01 |
| Syndecan-1, Month 4 (ng/ml) | 1.14 (0.8–1.63) | 0.462 | 0.21 | <0.01 |
| Syndecan-4, Day 1 (ng/ml) | 0.69 (0.47–1.01) | 0.056 | 0.14 | 0.02 |
| Syndecan-4, Month 4 (ng/ml) | 0.72 (0.53–0.98) | **0.034** | 0.11 | 0.02 |
| GDF-15, Day 1 (ng/ml) | 1.39 (0.89–2.16) | 0.150 | 0.31 | <0.01 |
| GDF-15, Month 4 (ng/ml) | 1.11 (0.75–1.63) | 0.609 | 0.22 | <0.01 |
| Periostin, Day 1 (ng/ml) | 0.998 (0.994–1.003) | 0.48 | <0.01 | <0.01 |
| Periostin, Month 4 (ng/ml) | 1 (0.996–1.003) | 0.796 | <0.01 | <0.01 |
| Osteopontin, Day 1 (ng/ml) | 0.998 (0.97–1.03) | 0.905 | 0.02 | <0.01 |
| Osteopontin, Month 4 (ng/ml) | 0.99 (0.95–1.02) | 0.446 | 0.02 | <0.01 |

*Univariable logistic regression analyses on dichotomized outcomes. A p-value <0.05 was considered significant and marked in bold.*

*GDF, growth differentiation factor; MVO, microvascular obstruction; EDV, end-diastolic volume; LVEF, left ventricular ejection fraction; MSI, myocardial salvage index.*

Table S3. Multivariable logistic regression analyses, model 1.

| **Biomarker** | **OR (95% CI)** | **p-value** | **Standard error** | **r^2^ (for the total model)** |
| --- | --- | --- | --- | --- |
| **MVO** | | | | |
| Syndecan-1, Day 1 (ng/ml) | 1.01 (0.72–1.41) | 0.955 | 0.17 | 0.29 |
| Syndecan-1, Month 4 (ng/ml) | 1.11 (0.73–1.7) | 0.624 | 0.24 | 0.29 |
| Syndecan-4, Day 1 (ng/ml) | 0.98 (0.65–1.47) | 0.924 | 0.2 | 0.29 |
| Syndecan-4, Month 4 (ng/ml) | 1.23 (0.86–1.76) | 0.26 | 0.22 | 0.29 |
| GDF-15, Day 1 (ng/ml) | 0.86 (0.52–1.41) | 0.548 | 0.22 | 0.29 |
| GDF-15, Month 4 (ng/ml) | 0.78 (0.44–1.37) | 0.385 | 0.23 | 0.29 |
| Periostin, Day 1 (ng/ml) | 1 (0.997–1.01) | 0.314 | <0.01 | 0.29 |
| Periostin, Month 4 (ng/ml) | 1 (0.997–1.004) | 0.681 | <0.01 | 0.29 |
| Osteopontin, Day 1 (ng/ml) | 0.99 (0.96–1.03) | 0.581 | 0.02 | 0.29 |
| Osteopontin, Month 4 (ng/ml) | 1.01 (0.98–1.05) | 0.391 | 0.02 | 0.29 |
| **Change in EDV >10%** | | | | |
| Syndecan-1, Day 1 (ng/ml) | 0.69 (0.51–0.94) | 0.02 | 0.11 | 0.1 |
| Syndecan-1, Month 4 (ng/ml) | 0.79 (0.55–1.14) | 0.211 | 0.15 | 0.08 |
| Syndecan-4, Day 1 (ng/ml) | 0.99 (­0.7–1.41) | 0.958 | 0.18 | 0.08 |
| Syndecan-4, Month 4 (ng/ml) | 0.71 (0.52–0.96) | **0.027** | 0.11 | 0.09 |
| GDF-15, Day 1 (ng/ml) | 1.1 (0.77–1.58) | 0.595 | 0.2 | 0.08 |
| GDF-15, Month 4 (ng/ml) | 1.41 (0.82–2.41) | 0.211 | 0.39 | 0.08 |
| Periostin, Day 1 (ng/ml) | 1 (0.998–1.01) | 0.31 | <0.01 | 0.08 |
| Periostin, Month 4 (ng/ml) | 1 (0.998–1.004) | 0.481 | <0.01 | 0.08 |
| Osteopontin, Day 1 (ng/ml) | 0.99 (0.96–1.02) | 0.719 | 0.02 | 0.08 |
| Osteopontin, Month 4 (ng/ml) | 1.03 (0.995–1.06) | 0.098 | 0.02 | 0.08 |
| **LVEF<50% at Month 4** | | | | |
| Syndecan-1, Day 1 (ng/ml) | 0.97 (0.69–1.37) | 0.858 | 0.17 | 0.35 |
| Syndecan-1, Month 4 (ng/ml) | 1.53 (0.98–2.38) | 0.062 | 0.35 | 0.37 |
| Syndecan-4, Day 1 (ng/ml) | 1.12 (0.71–1.79) | 0.625 | 0.27 | 0.35 |
| Syndecan-4, Month 4 (ng/ml) | 0.99 (0.67–1.45) | 0.946 | 0.19 | 0.36 |
| GDF-15, Day 1 (ng/ml) | 1.51 (0.78–2.9) | 0.226 | 0.51 | 0.36 |
| GDF-15, Month 4 (ng/ml) | 1.61 (0.86–3.01) | 0.137 | 0.51 | 0.37 |
| Periostin, Day 1 (ng/ml) | 1 (0.999–1.01) | 0.113 | <0.01 | 0.36 |
| Periostin, Month 4 (ng/ml) | 1 (0.999–1.01) | 0.108 | <0.01 | 0.37 |
| Osteopontin, Day 1 (ng/ml) | 0.99 (0.95–1.03) | 0.586 | 0.02 | 0.35 |
| Osteopontin, Month 4 (ng/ml) | 0.97 (0.92–1.03) | 0.367 | 0.03 | 0.36 |
| **Final infarct size >75th percentile** | | | | |
| Syndecan-1, Day 1 (ng/ml) | 1.1 (0.75–1.6) | 0.643 | 0.21 | 0.49 |
| Syndecan-1, Month 4 (ng/ml) | 0.99 (0.6–1.63) | 0.96 | 0.25 | 0.5 |
| Syndecan-4, Day 1 (ng/ml) | 0.94 (0.55–1.6) | 0.815 | 0.26 | 0.49 |
| Syndecan-4, Month 4 (ng/ml) | 0.83 (0.52–1.31) | 0.417 | 0.19 | 0.5 |
| GDF-15, Day 1 (ng/ml) | 1.03 (0.57–1.86) | 0.922 | 0.31 | 0.49 |
| GDF-15, Month 4 (ng/ml) | 0.84 (0.35–2.02) | 0.698 | 0.38 | 0.5 |
| Periostin, Day 1 (ng/ml) | 1 (0.99–1.003) | 0.355 | <0.01 | 0.5 |
| Periostin, Month 4 (ng/ml) | 1 (0.995–1.004) | 0.898 | <0.01 | 0.5 |
| Osteopontin, Day 1 (ng/ml) | 0.99 (0.94–1.04) | 0.589 | 0.03 | 0.49 |
| Osteopontin, Month 4 (ng/ml) | 0.99 (0.93–1.06) | 0.834 | 0.03 | 0.5 |
| **MSI<2/3** | | | | |
| Syndecan-1, Day 1 (ng/ml) | 0.9 (0.62–1.3) | 0.57 | 0.17 | 0.39 |
| Syndecan-1, Month 4 (ng/ml) | 0.62 (0.37–1.03) | 0.068 | 0.16 | 0.4 |
| Syndecan-4, Day 1 (ng/ml) | 0.54 (0.31–0.93) | 0.026 | 0.15 | 0.41 |
| Syndecan-4, Month 4 (ng/ml) | 0.71 (0.47–1.07) | 0.1 | 0.15 | 0.4 |
| GDF-15, Day 1 (ng/ml) | 0.8 (0.45–1.44) | 0.458 | 0.24 | 0.39 |
| GDF-15, Month 4 (ng/ml) | 0.59 (0.29–1.2) | 0.148 | 0.21 | 0.4 |
| Periostin, Day 1 (ng/ml) | 1 (0.99–1.006) | 0.895 | <0.01 | 0.39 |
| Periostin, Month 4 (ng/ml) | 1 (0.995–1.003) | 0.729 | <0.01 | 0.39 |
| Osteopontin, Day 1 (ng/ml) | 0.99 (0.95–1.03) | 0.599 | 0.02 | 0.39 |
| Osteopontin, Month 4 (ng/ml) | 1 (0.95–1.05) | 0.851 | 0.03 | 0.39 |

*Multivariable logistic regression analyses on dichotomized outcomes. A p-value <0.05 was considered significant and marked in bold. Model 1: Age, symptom-to-balloon time, post-conditioning status, peak troponin T.*

*GDF, growth differentiation factor; MVO, microvascular obstruction; EDV, end-diastolic volume; LVEF, left ventricular ejection fraction; MSI, myocardial salvage index.*

Table S4. Multivariable logistic regression analyses, model 2.

| **Biomarker** | **OR (95% CI)** | **p-value** | **Standard error** | **r^2^ (for the total model)** |
| --- | --- | --- | --- | --- |
| **MVO** | | | | |
| Syndecan-1, Day 1 (ng/ml) | 1.02 (0.73–1.44) | 0.893 | 0.18 | 0.29 |
| Syndecan-1, Month 4 (ng/ml) | 1.11 (0.72–1.7) | 0.630 | 0.24 | 0.29 |
| Syndecan-4, Day 1 (ng/ml) | 0.92 (0.6–1.39) | 0.687 | 0.2 | 0.29 |
| Syndecan-4, Month 4 (ng/ml) | 1.23 (0.85–1.78) | 0.261 | 0.23 | 0.29 |
| GDF-15, Day 1 (ng/ml) | 0.88 (0.54–1.43) | 0.601 | 0.22 | 0.29 |
| GDF-15, Month 4 (ng/ml) | 0.91 (0.48–1.74) | 0.780 | 0.3 | 0.29 |
| Periostin, Day 1 (ng/ml) | 1 (0.997–1.01) | 0.519 | <0.01 | 0.29 |
| Periostin, Month 4 (ng/ml) | 1 (0.997–1.004) | 0.708 | <0.01 | 0.29 |
| Osteopontin, Day 1 (ng/ml) | 0.99 (0.95–1.02) | 0.463 | 0.02 | 0.29 |
| Osteopontin, Month 4 (ng/ml) | 1.01 (0.98–1.05) | 0.364 | 0.02 | 0.29 |
| **Change in EDV >10%** | | | | |
| Syndecan-1, Day 1 (ng/ml) | 0.65 (0.46–0.91) | 0.012 | 0.11 | 0.14 |
| Syndecan-1, Month 4 (ng/ml) | 0.62 (0.41–0.94) | 0.025 | 0.13 | 0.13 |
| Syndecan-4, Day 1 (ng/ml) | 0.94 (0.65–1.36) | 0.749 | 0.18 | 0.11 |
| Syndecan-4, Month 4 (ng/ml) | 0.76 (0.56–1.05) | 0.093 | 0.12 | 0.12 |
| GDF-15, Day 1 (ng/ml) | 0.96 (0.59–1.56) | 0.874 | 0.24 | 0.11 |
| GDF-15, Month 4 (ng/ml) | 0.92 (0.51–1.64) | 0.769 | 0.27 | 0.12 |
| Periostin, Day 1 (ng/ml) | 1 (0.996–1.005) | 0.926 | <0.01 | 0.11 |
| Periostin, Month 4 (ng/ml) | 1 (0.995–1.002) | 0.445 | <0.01 | 0.12 |
| Osteopontin, Day 1 (ng/ml) | 0.99 (0.96–1.02) | 0.626 | 0.02 | 0.11 |
| Osteopontin, Month 4 (ng/ml) | 1.02 (0.99–1.06) | 0.145 | 0.02 | 0.12 |
| **LVEF<50% at Month 4** | | | | |
| Syndecan-1, Day 1 (ng/ml) | 0.94 (0.66–1.36) | 0.758 | 0.18 | 0.39 |
| Syndecan-1, Month 4 (ng/ml) | 1.3 (0.78–2.16) | 0.309 | 0.34 | 0.41 |
| Syndecan-4, Day 1 (ng/ml) | 1.01 (0.62–1.66) | 0.959 | 0.26 | 0.39 |
| Syndecan-4, Month 4 (ng/ml) | 1.1 (0.72–1.66) | 0.681 | 0.23 | 0.4 |
| GDF-15, Day 1 (ng/ml) | 1.57 (0.73–3.36) | 0.246 | 0.61 | 0.4 |
| GDF-15, Month 4 (ng/ml) | 0.99 (0.47–2.1) | 0.986 | 0.38 | 0.4 |
| Periostin, Day 1 (ng/ml) | 1 (0.996–1.006) | 0.733 | <0.01 | 0.39 |
| Periostin, Month 4 (ng/ml) | 1 (0.995–1.004) | 0.813 | <0.01 | 0.4 |
| Osteopontin, Day 1 (ng/ml) | 0.99 (0.94–1.04) | 0.626 | 0.02 | 0.39 |
| Osteopontin, Month 4 (ng/ml) | 0.95 (0.89–1.01) | 0.121 | 0.03 | 0.41 |
| **Final infarct size >75th percentile** | | | | |
| Syndecan-1, Day 1 (ng/ml) | 1.02 (0.69–1.5) | 0.914 | 0.2 | 0.49 |
| Syndecan-1, Month 4 (ng/ml) | 0.88 (0.52–1.48) | 0.622 | 0.23 | 0.5 |
| Syndecan-4, Day 1 (ng/ml) | 0.98 (0.57–1.71) | 0.956 | 0.28 | 0.49 |
| Syndecan-4, Month 4 (ng/ml) | 0.83 (0.52–1.34) | 0.452 | 0.2 | 0.5 |
| GDF-15, Day 1 (ng/ml) | 1.02 (0.51–2.07) | 0.953 | 0.37 | 0.49 |
| GDF-15, Month 4 (ng/ml) | 0.67 (0.27–1.63) | 0.374 | 0.3 | 0.5 |
| Periostin, Day 1 (ng/ml) | 1 (0.99–1.003) | 0.368 | <0.01 | 0.5 |
| Periostin, Month 4 (ng/ml) | 1 (0.99–1.004) | 0.759 | <0.01 | 0.5 |
| Osteopontin, Day 1 (ng/ml) | 0.99 (0.94–1.04) | 0.748 | 0.03 | 0.5 |
| Osteopontin, Month 4 (ng/ml) | 0.99 (0.93–1.05) | 0.696 | 0.03 | 0.5 |
| **MSI<2/3** | | | | |
| Syndecan-1, Day 1 (ng/ml) | 0.88 (0.61–1.28) | 0.509 | 0.17 | 0.38 |
| Syndecan-1, Month 4 (ng/ml) | 0.59 (0.35–0.998) | 0.049 | 0.16 | 0.4 |
| Syndecan-4, Day 1 (ng/ml) | 0.54 (0.31–0.94) | 0.029 | 0.15 | 0.4 |
| Syndecan-4, Month 4 (ng/ml) | 0.69 (0.45–1.07) | 0.102 | 0.15 | 0.39 |
| GDF-15, Day 1 (ng/ml) | 0.76 (0.42–1.37) | 0.356 | 0.23 | 0.38 |
| GDF-15, Month 4 (ng/ml) | 0.47 (0.24–0.91) | 0.026 | 0.16 | 0.4 |
| Periostin, Day 1 (ng/ml) | 1 (0.99–1.006) | 0.857 | <0.01 | 0.38 |
| Periostin, Month 4 (ng/ml) | 1 (0.995–1.004) | 0.774 | <0.01 | 0.38 |
| Osteopontin, Day 1 (ng/ml) | 0.99 (0.95–1.03) | 0.587 | 0.02 | 0.38 |
| Osteopontin, Month 4 (ng/ml) | 0.99 (0.94–1.05) | 0.842 | 0.03 | 0.38 |

*Multivariable logistic regression analyses on dichotomized outcomes. A p-value <0.05 was considered significant and marked in bold. Model 2: Age, symptom-to-balloon time, post-conditioning status, peak troponin T, NT-proBNP at month 4.*

*GDF, growth differentiation factor; MVO, microvascular obstruction; EDV, end-diastolic volume; LVEF, left ventricular ejection fraction; MSI, myocardial salvage index.*

Table S5. Univariable Cox proportional hazards model between biomarker levels and all-cause mortality

| **Univariable** | | | |
| --- | --- | --- | --- |
| **Biomarker** | **HR (95% CI)** | **p-value** | **Standard error** |
| GDF-15, Day 1 | 1.23 (0.93–1.64) | 0.149 | 0.18 |
| GDF-15, Month 4 | 1.59 (1.28–1.97) | <0.001 | 0.17 |
| Periostin, Day 1 | 1.006 (1.002–1.01) | 0.009 | <0.01 |
| Periostin, Month 4 | 1.007 (1.003–1.01) | 0.001 | <0.01 |
| Osteopontin, Day 1 | 1.02 (0.98–1.06) | 0.328 | 0.02 |
| Osteopontin, Month 4 | 0.99 (0.92–1.06) | 0.804 | 0.04 |
| Syndecan-1, Day 1 | 1.1 (0.75–1.62) | 0.609 | 0.22 |
| Syndecan-1, Month 4 | 1.5 (0.94–2.39) | 0.086 | 0.36 |
| Syndecan-4, Day 1 | 0.62 (0.36–1.07) | 0.087 | 0.17 |
| Syndecan-4, Month 4 | 0.48 (0.28–0.84) | 0.010 | 0.14 |

*Univariable Cox proportional hazards model. All concentrations in ng/ml. Hazard ratios are calculated for per 1 unit increase in concentration. P<0.05 were considered significant and marked in bold.*

*GDF, growth differentiation factor; NT-proBNP, NT-proBNP, N-terminal pro-B-type natriuretic peptide.*

Table S6. Mutivariable variable Cox proportional hazards model, model 1, between biomarker levels and all-cause mortality

| **Model 1** | | | |
| --- | --- | --- | --- |
| **Biomarker** | **HR (95% CI)** | **p-value** | **Standard error** |
| GDF-15, Day 1 | 1.08 (0-77–1.52) | 0.644 | 0.19 |
| GDF-15, Month 4 | 1.82 (1.36–2.43) | <0.0001 | 0.27 |
| Periostin, Day 1 | 1.005 (1.0004–1.01) | 0.034 | <0.01 |
| Periostin, Month 4 | 1.006 (1.002–1.01) | 0.003 | <0.01 |
| Osteopontin, Day 1 | 1.02 (0.98–1.06) | 0.364 | 0.02 |
| Osteopontin, Month 4 | 0.99 (0.92–1.07) | 0.832 | 0.04 |
| Syndecan-1, Day 1 | 1.11 (0.75–1.65) | 0.607 | 0.23 |
| Syndecan-1, Month 4 | 1.64 (1.01–2.67) | 0.044 | 0.41 |
| Syndecan-4, Day 1 | 0.74 (0.42–1.29) | 0.286 | 0.21 |
| Syndecan-4, Month 4 | 0.48 (0.26–0.88) | 0.017 | 0.15 |

*Multivariable Cox proportional hazards model. All concentrations in ng/ml. Hazard ratios are calculated for per 1 unit increase in concentration. Model 1: Age, symptom-to-balloon time, post-conditioning status, peak troponin T. P<0.05 were considered significant and marked in bold.*

*GDF, growth differentiation factor; NT-proBNP, NT-proBNP, N-terminal pro-B-type natriuretic peptide.*

Table S7. Mutivariable variable Cox proportional hazards model, model 1, between biomarker levels and all-cause mortality

| **Model 2** | | | |
| --- | --- | --- | --- |
| **Biomarker** | **HR (95% CI)** | **p-value** | **Standard error** |
| GDF-15, Day 1 | 0.66 (0.42–1.05) | 0.079 | 0.16 |
| GDF-15, Month 4 | 1.45 (1.000–2.11) | 0.050 | 0.28 |
| Periostin, Day 1 | 1.003 (0.998–1.008) | 0.276 | <0.01 |
| Periostin, Month 4 | 1.005 (1.0007–1.01) | 0.024 | <0.01 |
| Osteopontin, Day 1 | 1.02 (0.96–1.08) | 0.543 | 0.03 |
| Osteopontin, Month 4 | 0.97 (0.9–1.04) | 0.424 | 0.04 |
| Syndecan-1, Day 1 | 0.95 (0.53–1.7) | 0.86 | 0.28 |
| Syndecan-1, Month 4 | 1.27 (0.75–2.18) | 0.374 | 0.35 |
| Syndecan-4, Day 1 | 0.50 (0.23–1.08) | 0.079 | 0.2 |
| Syndecan-4, Month 4 | 0.44 (0.22–0.84) | 0.014 | 0.15 |

*Multivariable Cox proportional hazards model. All concentrations in ng/ml. Hazard ratios are calculated for per 1 unit increase in concentration. Model 2: model 1 + NT-proBNP at Month 4. P<0.05 were considered significant and marked in bold.*

*GDF, growth differentiation factor; NT-proBNP, NT-proBNP, N-terminal pro-B-type natriuretic peptide.*

Table S8. AUC for biomarkers and total mortality

| Biomarker | AUC |
| --- | --- |
| All-cause mortality | |
| GDF-15, Month 4 | 0.86 |
| Periostin, Day 1 | 0.60 |
| Periostin, Month 4 | 0.66 |
| Syndecan-4, Month 4 | 0.72 |
| MVO | |
| Syndecan-1, Day 1 | 0.60 |
| Syndecan-1, Month 4 | 0.62 |
| Change in EDV>10% | |
| Syndecan-4, Month 4 | 0.43 |
| LVEF<50% at Month 4 | |
| GDF-15, Day 1 | 0.63 |
| GDF-15, Month 4 | 0.58 |
| Syndecan-1, Month 4 | 0.64 |
| Final infarct size >75th percentile | |
| GDF-15, Day 1 | 0.64 |
| Syndecan-1, Day 1 | 0.62 |
| Syndecan-1, Month 4 | 0.62 |
| MSI>1/3 | |
| Syndecan-4, Day 1 | 0.57 |
| Syndecan-4, Month 4 | 0.59 |

*AUC for biomarkers with significant regression results. MVO, microvascular obstruction; EDV, end-diastolic volume; LVEF, left ventricular ejection fraction; MSI, myocardial salvage index.*

Fig S1. Heatmap correlations between biomarkers and continuous outcomes.

*Heatmap displaying spearman’s rho correlation coefficient results. Colours in the heatmap reflect the value of the coefficient according to the panel to the right of the heatmap.*

*GDF, growth differentiation factor; LVEF, left ventricular ejection fraction; EDV, end-diastolic volume; MSI, myocardial salvage index; TnT, troponin T.*
